# A Novel Photoelectrochemical Biosensor for Tyrosinase and Thrombin Detection

**DOI:** 10.3390/s16010135

**Published:** 2016-01-21

**Authors:** Jiexia Chen, Yifan Liu, Guang-Chao Zhao

**Affiliations:** 1 Anhui Key Laboratory of Chem-Biosensing, School of Chemistry and Materials Science, Anhui Normal University, Wuhu 241000, China; jiexchen@163.com (J.C.); lyffxk@126.com (Y.L.); 2 Departement of Chemistry, Wannan Medical College, Wuhu 241002, China

**Keywords:** tyrosinase, thrombin, peptide, photoelectrochemical biosensor

## Abstract

A novel photoelectrochemical biosensor for step-by-step assay of tyrosinase and thrombin was fabricated based on the specific interactions between the designed peptide and the target enzymes. A peptide chain with a special sequence which contains a positively charged lysine-labeled terminal, tyrosine at the other end and a cleavage site recognized by thrombin between them was designed. The designed peptide can be fixed on surface of the CdTe quantum dots (QDs)-modified indium-tin oxide (ITO) electrode through electrostatic attraction to construct the photoelectrochemical biosensor. The tyrosinase target can catalyze the oxidization of tyrosine by oxygen into ortho-benzoquinone residues, which results in a decrease in the sensor photocurrent. Subsequently, the cleavage site could be recognized and cut off by another thrombin target, restoring the sensor photocurrent. The decrease or increase of photocurrent in the sensor enables us to assay tyrosinase and thrombin. Thus, the detection of tyrosinase and thrombin can be achieved in the linear range from 2.6 to 32 μg/mL and from 4.5 to 100 μg/mL with detection limits of 1.5 μg/mL and 1.9 μg/mL, respectively. Most importantly, this strategy shall allow us to detect different classes of enzymes simultaneously by designing various enzyme-specific peptide substrates.

## 1. Introduction

In recent years, the newly developed photoelectrochemical (PEC) detection method has been a promising analytical method for biological assays [1,2,3,4]. Benefiting from the separation of excitation source and detection signals, photoelectrochemical sensors are very sensitive and display low background signals. The photocurrent generation mechanism is based on the photoexcitation of photoactive materials that leads to the transfer of electrons from the valence band to the conduction band, thus forming electron-hole pairs. Then the ejection of the conduction-band electrons to the electrode, with the concomitant transfer of electrons from an electron donor (or hole scavenger) present in solution, yields an anodic photocurrent [5]. Therefore, while previously the sensing principle of PEC mainly focused on the corresponding photocurrent signal changes produced by holes reduction caused by analytes acting as electron donors, a series of photoelectrochemical platforms have been developed to detect dopamine [6], glutathione (GSH) [7], nicotinamide adenine dinucleotide [8] and so on. All of these works are based on changing the direct electron transfer process between the ambient environment and the photoactive materials before and after the biorecognition events. Despite these extensive investigations, how to exploit an innovative PEC platform that could offer a new and common avenue for future PEC analysis is still a challenge. In this paper we propose a new strategy using a peptide as an intermediate that can be catalyzed by different enzymes. Then the electron transfer from the conduction band of the photoactive material to the electrode was changed indirectly. We can achieve a photocurrent signal diminution or enhancement, so as to indirectly realize the determination of different types of proteases. 

Proteases have been researched widely because they are involved in various diseases such as AIDS, cancer, inflammation, and neurodegenerative diseases [9,10,11]. Typical examples include metalloproteinases, serine proteases, thiol proteases and carboxyl proteases. Tyrosinase, as a Cu(II)-containing monooxygenase, is one of the most important metalloproteinases and plays a key role for the biosynthesis of natural pigment melanins [10]. In the presence of oxygen, it catalyzes the oxidation of tyrosine via L-3, 4-dihydroxyphenylalanine (l-DOPA) to dopaquinone. Then eumelanin is formed through spontaneous auto-oxidation and polymerization [12]. Elevated amounts of tyrosinase are found in melanoma cancer cells, and the enzyme is viewed as an indicative marker for this type of malignant cell [10]. Thus the development of effective methods for the quantitative detection of the catalytic activity of tyrosinase is very important. So far, many techniques have been exploited to detect tyrosinase, such as liquid chromatography and gel electrophoresis [13], fluorescence [14,15,16], electrochemical, photoelectrochemical, and piezoelectric method [17], but most of them have limitations in a multiplexed assay.

Similarly, thrombin acts as a serine protease that not only converts soluble fibrinogen into insoluble fibrin, but also catalyzes many other coagulation-related reactions. Thrombin is involved in pathological conditions involving central nervous system injuries, thromboembolic diseases, and Alzheimer’s disease [18,19]. Therefore, to exploit simple and highly sensitive methods for thrombin detection is very important for both clinical practice and diagnostic applications. In recent years, aptamers have been applied to a series of thrombin detection methods such as fluorescence [20,21,22], photoluminescence [23], electrochemical [24,25,26], and photoelectrochemical analysis method [27] and so on. All of those methods are founded on the structural change from a single-stranded aptamer to a quadruplex structure caused by thrombin. However, they ignore the fact that thrombin is a protease that can be used to selectively cleave the amide bond between arginine and glycine residues with specificity, and there are only a few reports using this property for the detection of thrombin [28,29]. Thus development of a high sensitive method in a multiplexed manner for protease monitoring is still of great interest for the diagnosis of protease-relevant diseases and exploitation of potential drugs. 

Considering the significance of sensing proteases, herein, we tried to develop a photoelectrochemical sensor to detect proteases using tyrosinase and thrombin as model enzymes. This strategy is based on the enzymes’ catalytic activity toward a designed peptides modified on the surface of electrodes to change the electrode structure. Thus, the electron transfer process is changed, which will result in a photocurrent signal change. The proposed sensing strategy allows us to develop a time-saving, miniaturized instrument and simple operation method to assay two kinds of model enzymes. We hope that this study may serve as a foundation to develop new photoelectrochemical biosensors for different classes of proteases by designing various enzyme-specific peptide substrates. 

## 2. Materials and Methods 

### 2.1. Reagents and Instruments

Cadmium chloride (CdCl_2_·2.5H_2_O), sodium metaborate (NaBH_4_) and sodium hydroxide were purchased from Shanghai Chemical Reagent Co. (Shanghai, China). Ascorbic acid (AA), poly(diallyldimethylammonium chloride) PDDA (20%, w/w in water, molecular weight 200 000-350 000), 3-mercaptopropionic acid (MPA) , Te powder were all obtained from Aladdin Chemistry Co. Ltd. (Shanghai, China). Tyrosinase (from mushroom) and thrombin (from bovine serum) were both obtained from Sigma-Aldrich (Natick, MA, USA). The designed Cys-Ser-Ala-Phe-Pro-Arg-Gly-Arg-Tyr peptide was synthesized by Apeptide Co., Ltd. (Shanghai, China) and its purity (by HPLC) was more than 99%. Dulbecco’s phosphate buffer (PBS, pH 7.4, 10 mM) was used to prepare the solution of peptide and QDs. PBS (pH 6.8, 10 mM) was used for incubating tyrosinase and PBS (pH 8.3, 10 mM) was used for incubating thrombin. The photocurrent assay was carried out in PBS (pH 7.4, 0.1 M) including 0.1 M ascorbic acid (AA) which was served as a sacrificial electron donor. All other reagents were of analytical grade and were used without further purification. Double distilled water (18 MΩ·cm^−1^), which was obtained from a Milli-Q water purification system, was used in the preparation of all aqueous solutions. 

The ITO slice (STN type, ITO coating 30 ± 5 nm, and sheet resistance ≤ 10 Ω/square) was obtained from Wuhu Token Sciences Co., Ltd, (Wuhu, China). Photoelectrochemical detections were performed with a home-built photoelectrochemical system. A 500 W Xe lamp equipped with a monochromator served as the visible light source. The monochromatic illuminating light intensity was about 400 µW/cm^2^, which estimated with a radiometer (Photoelectric Instrument Factory of Beijing Normal University, Beijing, China). Photocurrent was measured on a 660b electrochemical workstation (CHI, Shanghai, China). The measurements were based on a conventional three-electrode system with a CdTe QD-modified ITO electrode with an area of 0.25 cm^2^ as the working electrode, a platinum wire as the auxiliary electrode and a saturated Ag/AgCl electrode as the reference electrode. All the photocurrent measurements were performed at a constant potential of −0.5 V (*vs.* saturated Ag/AgCl) with a 0.1 M PBS (pH 7.4) solution including 0.1 M AA as the supporting electrolyte. Electrochemical impedance spectroscopy (EIS) was performed in 5.0 mM K_3_[Fe(CN)_6_]/K_4_[Fe(CN)_6_] (1:1) mixture containing 0.1 M KCl as a redox probe over a frequency ranging from 0.1 Hz to 100 kHz at an applied voltage of 5 mV with the CHI 660b electrochemical workstation.

### 2.2. Preparation of Water Soluble CdTe QDs

MPA (3-mercaptopropionic acid)-stabilized CdTe QDs were synthesized according to the method described in our previous work [30]. Briefly, CdCl_2_·2.5H_2_O (0.15 mmol), and MPA (32 μL) were dissolved into doubly distilled water (50 mL) in a three-neck flask to form the cadmium precursor. The pH of mixture was adjusted to 11 with 1 M NaOH solution, and stirred in an Ar atmosphere which was deaerated for 30 min. Subsequently, freshly prepared NaHTe aqueous solution (0.2 mL), which was prepared by reaction of NaBH_4_ (40 mg) and Te powder (50 mg) in distilled water (1 mL) at 26 °C for 1 h, was injected into the above solution under stirring to obtain MPA-capped water-soluble CdTe QDs. Finally, the reaction mixture was refluxed for 8 h in an Ar atmosphere. The crude product was washed with ethanediol and centrifuged to remove excess precursors. The obtained MPA-CdTe QDs were dispersed in water and stored at 4 °C for future use.

### 2.3. Preparation of Modified Electrodes

We fixed QDs on the ITO electrode surface by the layer-by-layer self-assembly technology. Firstly, the ITO slices were cleaned by successive immersion with sonication in ammonia, doubly distilled water, ethanol, doubly distilled water and ethanol/1 M NaOH (v/v, 1:1), respectively, for about 15 min each. Then, the CdTe-modified electrode was fabricated by alternately immersing the cleaned ITO slices into a solution of 2% PDDA including 0.5 M NaCl and the QDs solution for 15 min, respectively. This process was repeated three times to obtain desired the photocurrent intensity (this electrode is denoted as ITO/QDs). The ITO slices were carefully washed with doubly distilled water after each dipping step. Finally, the as-obtained ITO slices was incubated in 0.5 mL 0.01 M PBS at pH 7.4 including 0.02 mg/mL peptide for 2 h at room temperature to immobilize the peptide (denoted as ITO/QDs/peptide). The films were carefully washed with washing buffer solution and doubly distilled water after the assembly step to remove excess peptide chains. The obtained ITO slices were used as the working electrode and stored at 4 °C in a dark environment before use.

### 2.4. PEC Detection

For the detection of tyrosinase, the ITO/QDs/peptide device was used as the working electrode. The electrodes were immersed in various concentrations of tyrosinase solution and incubated for 35 min. After incubation, the photocurrent responses of the electrodes were recorded. A signal-off of the electrode with concentrations of tyrosinase was obtained. After completing the determination of tyrosinase, the sensor can be used directly for the determination of thrombin. The electrodes (after incubation in 32 μg/mL tyrosinase solution, noted as ITO/QDs/peptide/tyrosinase) were incubated in different concentrations of thrombin for 60 min. The photocurrent responses of the electrode were measured under the same conditions. A signal-on of the electrode with concentrations of thrombin was obtained. Therefore, the stepwise detection of tyrosinase and thrombin can be realized using a PEC biosensor.

If wanting to detect thrombin alone, the ITO/QDs/peptide/tyrosinase should be used as working electrode. However, the fabricated PEC biosensor cannot be used for the simultaneous detection of tyrosinase and thrombin when they are presented in the same solution. As far as we know, however, there are no diseases that require measuring the concentrations of these two enzymes at the same time. All of the above photoelectrochemical measurements were performed in 0.1 M PBS (pH 7.4) including 0.1 M AA which was acted as a sacrificial electron donor with 400 nm light on and off. The applied potential was −0.5 V.

## 3. Results and Discussion

### 3.1. Construction of the PEC Enzymes Sensor

**Scheme 1:**
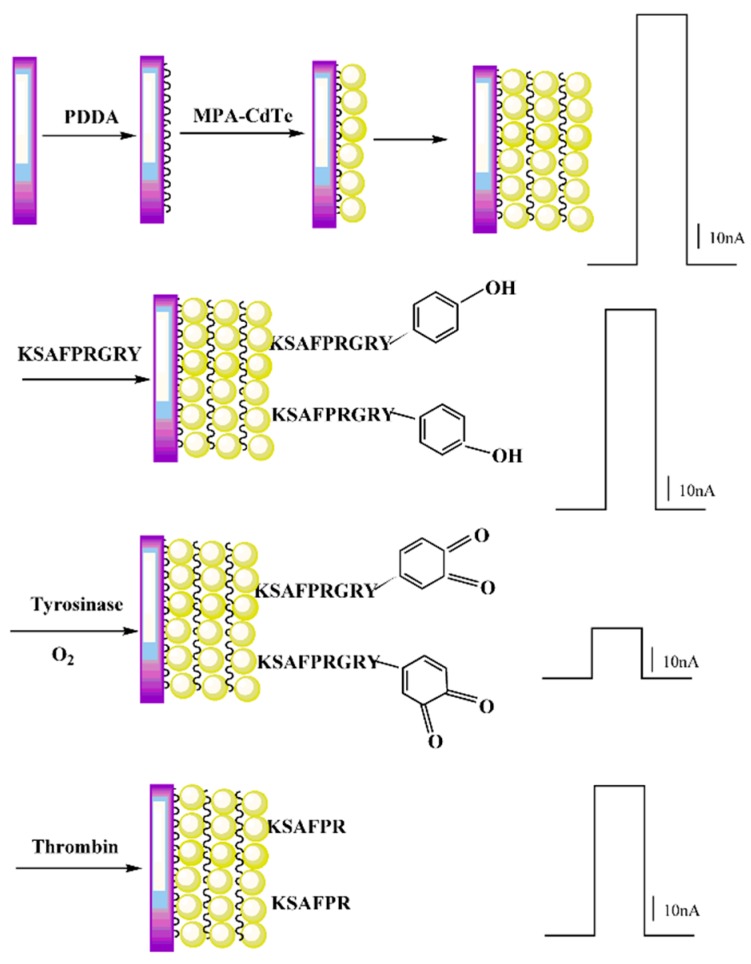
Schematic illustration of the stepwise construction and detection process for the determination of the enzymes.

The fabrication process and detection mechanism of the sensing strategy are shown in Scheme 1. Firstly, we fixed QDs on the ITO electrode surface by a standard layer-by-layer self-assembly technology. Upon light irradiation, the QD-modified ITO electrode showed a satisfactory photocurrent. Then, the designed peptide which contains a positively charged lysine-labeled terminal could be attached to the negatively charged QDs through electrostatic attraction. The other end of the peptide was tyrosine, which contains phenolic hydroxyl groups. In the presence of O_2,_ tyrosinase catalyzes the oxidation of tyrosine to produce l-DOPA that is subsequently converted to *o*-quinone [12]. 

It had been reported that quinones are the oxidized form of aromatic diol molecules and are partially or fully transformed into the reduced forms. For this reason, *o*-quinone is frequently found as an electron-acceptor in electron transfer systems in biology, chemistry, and industrial fields [31]. The generated *o*-quinone residues have been used as quenchers of the fluorescence of the QDs [32]. In the PEC process, the *o*-quinone products were expected to act as quenchers of the photocurrent of the QDs and thus provided a path for the PEC detection of tyrosinase [32,33], so the tyrosinase concentrations were indirectly measured through the decrease in photocurrent intensity resulting from the specific enzyme-catalyzed oxidation. Finally, after incubating in thrombin, since the designed peptide includes Arg-Gly bonds that can be selectively cleaved by thrombin [28,32], the *o*-quinone units will thus leave the electrode surface following the part of peptide hydrolytically cleaved by thrombin. The photocurrent of the QDs is anticipated to be restored, so the thrombin concentrations were indirectly measured through the recovery of photocurrent intensity. By integrating the enzyme-catalyzed oxidation effect of tyrosinase and the enzyme hydrolysis effect of thrombin in an analytical strategy, tyrosinase and thrombin could be sensitively detected. Furthermore, the proposed strategy can be extended for the development of other enzyme-based PEC biosensors.

### 3.2. Characterization of the Sensor

**Figure 1:**
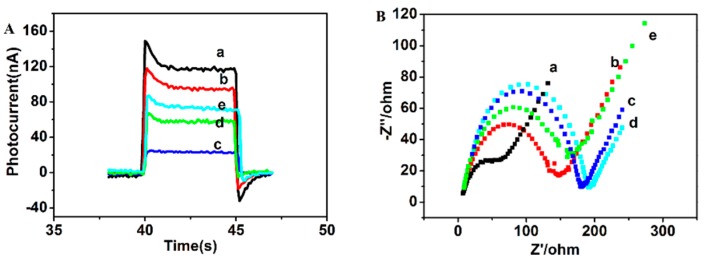
(**A**) Photocurrent response for (a) ITO/QDs, (b) ITO/QDs/peptide, (c) ITO/QDs/ peptide/tyrosinase (32 μg/mL), (d) ITO/QDs/peptide/tyrosinase (32 μg/mL)/thrombin (25 μg/mL ), (e) ITO/QDs/peptide/tyrosinase (32 μg/mL)/thrombin (50 μg/mL) in 0.1 M PBS (pH 7.4) containing 0.1 M AA at –0.5 V to a light excitation at 400 nm; (**B**) EIS of the modified electrodes in 0.1 M KCl containing 5 mM [Fe(CN)_6_]^3−^/[Fe(CN)_6_]^4−^ (1:1): (a) ITO, (b) ITO/QDs, (c) ITO/QDs/peptide, (d) ITO/QDs/peptide/tyrosinase (32 μg/mL), (e) ITO/QDs/peptide/tyrosinase (32 μg/mL)/thrombin (50 μg/mL). EIS were recorded between 0.01 Hz to 100 kHz with applied voltage of 5 mV.

The sensor preparation process was characterized by photoelectrochemical measurements (PEC) and electrochemical impedance spectroscopy (EIS). Figure 1A depicts the photocurrent responses of different modified electrodes during the fabrication process. After assembling of (PDDA/QDs)_3_ on the surface of the ITO electrode, the photocurrent increased prominently (curve a). Photoexcitation of these CdTe QDs yielded electron-hole pairs. In the presence of ascorbic acid (AA), as the employed electron donor, the valence band holes oxidize AA, while the conduction band electrons are injected into the electrode to give rise to the photocurrent [34]. 

**Figure 2:**
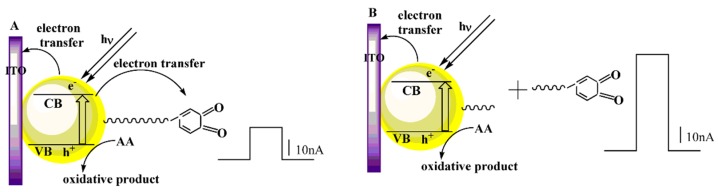
Electron transfer mechanism after incubating in (**A**) tyrosinase and (**B**) thrombin.

After the peptide immobilization on the electrode surface, the photocurrent still maintained about 82% of the original intensity (curve b). This may be due to the increased steric hindrance caused by the immobilized peptide partially blocking the diffusion of AA to the surface of CdTe for hole scavenging [35,36]. The photocurrent decreased greatly after the prepared modified electrodes were immersed in tyrosinase solution and incubated for 35 min (curve c). This phenomenon could be explained by the fact that in addition to the ejection of the conduction-band electrons to the electrode, more of the electrons were transferred to *o*-quinone, which resulted in a photocurrent decrease. The electron transport process mentioned above can be visualized as in Figure 2A. Subsequently, the electrode was incubated in thrombin solution and its photocurrent intensity was restored (curves d and e). These are attributed to the *o*-quinone removal from the electrode surface. The electron transport as illustrated in Figure 2A was damaged, and the conduction band electrons of the quantum dots were injected again into the electrode. The photocurrent generation mechanism can be described as in Figure 2B. Figure 1B showed the EIS of different modified electrodes. Compared with bare ITO electrode (curve a), the diameter of the low frequency semicircle increased after the assembling of (PDDA/QDs)_3_ on the surface of the ITO electrode (curve b), which could be attributed to the electrostatic repulsion between the negatively charged [Fe(CN)_6_]^3−^/^4−^ redox probe and negatively charged CdTe QDs. This further suggested that the (PDDA/QDs) multilayer film was successfully assembled onto the electrode surface. Subsequently, the peptide immobilization on the multilayer film ocurred, and then the diameter increased further (curve c), which was ascribed to the increased steric hindrance effect of the peptide chain that would hinder the diffusion of the redox probe to the electrode [30]. By incubating in tyrosinase solution (with enough oxygen), almost no change was observed in the impedance (curve d). This suggested that the steric hindrance effect of the peptide still existed. After incubating in thrombin, the diameter decreased significantly (curve e), because the peptide on CdTe QDs was cleavaged by thrombin, and thus the steric hindrance effect decreased. This EIS result fully corresponds to the variation of photocurrent response as shown in Figure 1A and also further demonstrates that the PEC biosensor was successfully constructed as expected.

### 3.3. Optimization of Experimental Conditions

**Figure 3:**
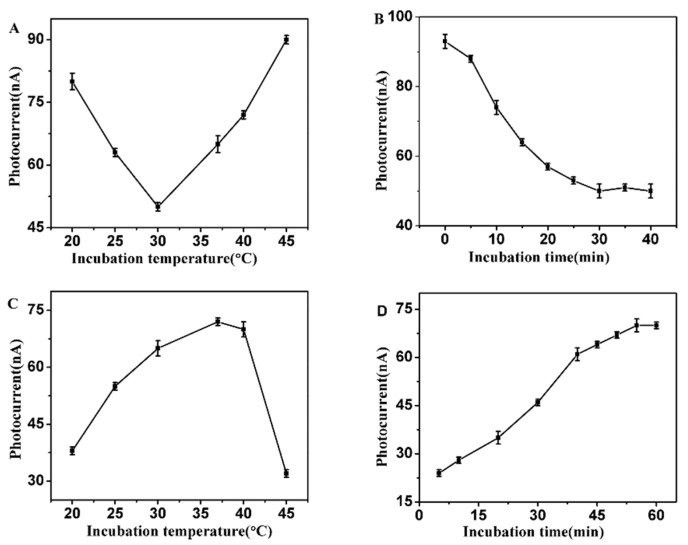
Effects of incubation temperature and time on photocurrent signals in presence of (**A**,**B**) 12.5 μg/mL tyrosinase; (**C**,**D**) 50 μg/mL thrombin. The PEC tests are measured in 0.1 M PBS (pH 7.4) containing 0.1 M AA at −0.5 V to a light excitation at 400 nm.

The incubation temperature and time play an important role in the performance of enzyme reactions. To achieve the best analysis performance, we optimized the relevant reaction conditions. Figure 3 shows the effects of incubation temperature and time on the photocurrent responses, respectively. As can be seen from Figure 3A, on increasing the temperature, there is a gradual decrease in the photocurrent intensity up to 30 °C, followed by a sharp increase in intensity in the 30–45 °C temperature range. The enzyme showed highest activity at temperature of 30 °C [37]. Therefore, 30 °C was chosen as the optimum incubation temperature for tyrosinase detection. For thrombin, as shown in Figure 3C, the maximum photocurrent response in the presence of thrombin layed in the range of 35 °C to 37 °C. The optimum temperature for thrombin was 37 °C. Figure 3B,D shows the effect of incubation time on the photocurrent response under the optimal incubation temperature conditions, respectively. The photocurrent decreased with increasing incubation time for tyrosinase, and reached a plateau in 30 to 40 min, indicating the peptides on the surface of the electrode were completely consumed. As a result, 35 min was selected as the optimal incubation time for tyrosinase. Compared with tyrosinase, the incubation time for thrombin of modified electrode was further optimized as showed in Figure 3D. The photocurrent recovered gradually with the increase in incubation time and then reached a platform at 50 to 60 min, showing that the reaction had reached equilibrium as well. Therefore, an optimal incubation time of 60 min for thrombin was selected for the subsequent PEC measurements.

### 3.4. PEC Biosensing of Tyrosinase and Thrombin

**Figure 4:**
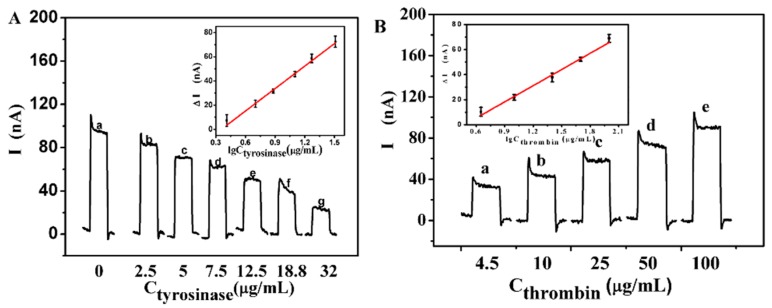
(**A**) Photocurrent response of the proposed sensor at 0, 2.5, 5, 7.5, 12.5, 18.8, 32 μg/mL tyrosinase (from a to g). Inset: calibration curve; (**B**) Photocurrent response of the proposed sensor at 4.5, 10, 25, 50, 100 μg/mL thrombin (from a to e). Inset: calibration curve. All the photocurrent responses were measured in 0.1 M PBS (pH 7.4) containing 0.1 M AA at −0.5 V to a light excitation at 400 nm.

The PEC biosensing principle was exploited to provide a way to quantify the targets. Tyrosinase and thrombin were selected as model enzymes and detected under optimal conditions by this PEC biosensor. Figure 4A shows the concentration dependence of the photocurrent signals of the designed biosensor under the optimal conditions. It was observed clearly that the photocurrent intensity decreased as tyrosinase concentrations increased till 32 μg/mL. The calibration plot of photocurrent intensity *versus* the logarithm of tyrosinase concentration in the range from 2.6 μg/mL to 32 μg/mL showed good linearity. If the concentration of tyrosinase increased over 40 μg/mL, then there was no further decrease in the photocurrent intensity, because of saturation of the enzyme substrate. The regression equation was ΔI (nA) = −21.99 + 61.94 logc_tyrosinase_ with a correlation coefficient of 0.991, where ΔI was the photocurrent of the ITO/QDs/peptide modified electrode before and after incubation with different concentrations of tyrosinase and c_tyrosinase_ is the concentration of tyrosinase (in μg/mL). A detection limit of 1.5 μg/mL can be estimated at 3σ. Compared with similar work reported in Table 1, our work showed satisfactory test results. This novel sensing strategy would open a new avenue for PEC detection of tyrosinase. 

The PEC biosensing concept can not only be used to determine the activity of tyrosinase, but also can be used for the determination of thrombin. The quantitative behavior of this photoelectrochemical enzyme sensor was assessed by measuring the dependence of the increased photocurrent intensity (ΔI) before and after incubation with different concentrations of thrombin. Results are shown in Figure 4B, where the photocurrent intensity gradually recovered with increasing thrombin concentrations up to 100 μg/mL at the optimal incubation temperature of 37 °C. The corresponding calibration curve is shown in the inset of Figure 4B. 

The increased value of the photocurrent intensity was linearly related to the concentration of thrombin on a logarithmic scale in a wide range from 4.5 to 100 μg/mL. The regression equation was ΔI (nA) = −20.51 + 43.12 logc_thrombin_ (μg/mL) with a regression coefficient of 0.988, where ΔI was the photocurrent of the ITO/QDs/peptide/tyrosinase (32 μg/mL)-modified electrode after and before incubation with different concentrations of thrombin. The lower detection limit can be estimated to be 1.9 μg/mL. Compared with the analytical performance of similar work reported as shown in Table 2, our work showed satisfactory test results. The above results indicated that the developed sensor showed a reasonable linear range and acceptable detection limits for the assay of tyrosinase and thrombin.

**Table 1:** Analytical performance of various tyrosinase assay methods.

Method	Linear Range	Detection Limit	Reference
Photoelectrochemical Method	2.6–32 μg/mL (1.3–16 U/mL)	1.5 μg/mL (0.8 U/mL)	This work
Photoelectrochemical Method	0.5–10 U/mL	0.1 U/mL	[17]
Electrochemical Method	0–10 U/mL	1 U/mL	[17]
Fluorescence Method	0–100 U/mL	n.s	[15]
Fluorescence Method	n.s	0.05 U/mL	[14]

**Table 2:** Analytical performance of various methods for thrombin.

Method	Linear Range	Detection Limit	Reference
Photoelectrochemical Method	4.5–100 μg/mL (0.12–2.7 nM)	1.9 μg/mL (50 pM)	This work
Fluorescence Method	0.5–10 pM	0.2 pM	[22]
Photoluminescence Method	0.02–200 nM	20 pM	[23]
Electrochemical Method	1 pM–25 nM	1 pM	[25]
Cleavage-Sensing Method	25 ng/mL–100 μg/mL	n.s.	[28]

### 3.5. Reproducibility, Specificity and Stability

**Figure 5:**
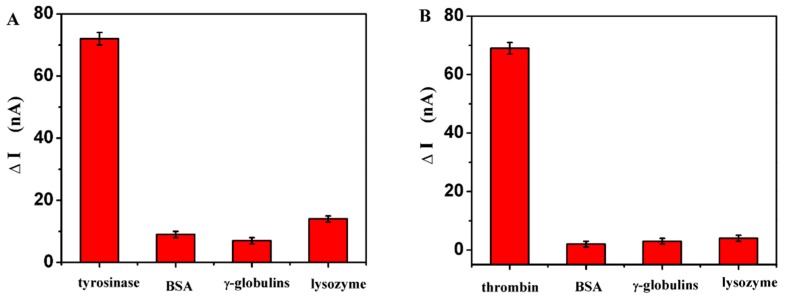
Selectivity of the proposed sensor to (**A**) tyrosinase and (**B**) thrombin by comparing it to the interfering proteins at the 60 μg/mL level and 120 μg/mL level: BSA, γ-globulin and lysozyme, respectively.

The reproducibility of the proposed PEC biosensor was estimated from the analysis of four freshly prepared biosensors. With tyrosinase at concentrations of 7.5 and 18.8 μg/mL, the PEC biosensor showed relative standard deviations of 5.5% and 7.2%, respectively. For thrombin at concentrations of 10 and 50 μg/mL, the PEC biosensor showed relative standard deviations of 6.4% and 7.0%, respectively. These results indicated that the biosensor had good fabrication reproducibility. To confirm the photocurrent change from the specificity of the target enzyme, and also to reveal the selectivity of this sensor, the as-prepared sensor was incubated with 60 μg/mL bovine serum albumin (BSA), gamma globulin and lysozyme as contrast experiments for tyrosinase. To compared with tyrosinase, the as-prepared sensor was incubated with 120 μg/mL bovine serum albumin (BSA), gamma globulin and lysozyme as thrombin contrast experiments, as shown in Figure 5. As can be seen, the ΔI of tyrosinase or thrombin was about 7 and 20 times higher than that of these protein molecules, respectively. These results demonstrated that the biosensor had satisfactory selectivity for the two target enzymes. The long-term stability of the sensor was also investigated. The photocurrent was very stable over time without any noticeable decrease for 100 μg/mL of thrombin during light on and off experiments which were repeated more than 10 times. The result indicated that the electrode had a good stability and did not show any significant change in photocurrent intensity after the modified electrode was stored in dark condition at 4 °C for two weeks. These results indicated that this biosensor has acceptable long term stability.

## 4. Conclusions

In conclusion, we have developed a novel photoelectrochemical biosensor for double enzyme detection. The photoelectrochemical platform not only can be used to detect tyrosinase by the signal diminution of the photocurrent, based on the catalytic oxidation of a peptide substrate, but also can use for detecting thrombin by the photocurrent signal enhancement based on the enzyme digestion reaction of thrombin. To the best of our knowledge, this is the first time anyone has made use of a single substrate for detecting two enzymes step-by-step. The proposed photoelectrochemical biosensor showed good performance in the monitoring of tyrosinase and thrombin with a wide linear range, low detection limits and high sensitivity. What is more important, this designed sensing strategy can be expanded to detect more enzymes by designing of suitable amino acid sequences for the peptide chains of substrates.
